# Professional advice for primary healthcare workers in Ethiopia: a social network analysis

**DOI:** 10.1186/s12913-020-05367-3

**Published:** 2020-06-17

**Authors:** Kate Sabot, Karl Blanchet, Della Berhanu, Neil Spicer, Joanna Schellenberg

**Affiliations:** 1 The Centre for Maternal, Adolescent, Reproductive and Child Health (MARCH), London School of Hygiene and Tropical Medicine, London, WC1E 7HT UK; 2 Department of Disease Control, Faculty of Infectious and Tropical Diseases, London School of Hygiene and Tropical Medicine, Keppel Street, London, WC1E 7HT UK; 3 Department of Global Health, Faculty of Public Health and Policy, London School of Hygiene and Tropical Medicine, London, WC1E 7HT UK

**Keywords:** Social network analysis, Healthcare workers, Professional advice, Advice networks, Maternal and newborn health, Ethiopia, Health extension workers, Primary care, Knowledge sharing

## Abstract

**Background:**

In an era of increasingly competitive funding, governments and donors will be looking for creative ways to extend and maximise resources. One such means can include building upon professional advice networks to more efficiently introduce, scale up, or change programmes and healthcare provider practices. This cross-sectional, mixed-methods, observational study compared professional advice networks of healthcare workers in eight primary healthcare units across four regions of Ethiopia. Primary healthcare units include a health centre and typically five satellite health posts.

**Methods:**

One hundred sixty staff at eight primary healthcare units were interviewed using a structured tool. Quantitative data captured the frequency of healthcare worker advice seeking and giving on providing antenatal, childbirth, postnatal and newborn care. Network and actor-level metrics were calculated including density (ratio of ties between actors to all possible ties), centrality (number of ties incident to an actor), distance (average number of steps between actors) and size (number of actors within the network). Following quantitative network analyses, 20 qualitative interviews were conducted with network study participants from four primary healthcare units. Qualitative interviews aimed to interpret and explain network properties observed. Data were entered, analysed or visualised using Excel 6.0, UCINET 6.0, Netdraw, Adobe InDesign and MaxQDA10 software packages.

**Results:**

The following average network level metrics were observed: density .26 (SD.11), degree centrality .45 (SD.08), distance 1.94 (SD.26), number of ties 95.63 (SD 35.46), size of network 20.25 (SD 3.65). Advice networks for antenatal or maternity care were more utilised than advice networks for post-natal or newborn care. Advice networks were typically limited to primary healthcare unit staff, but not necessarily to supervisors. In seeking advice, a colleague’s level of training and knowledge were valued over experience. Advice exchange primarily took place in person or over the phone rather than over email or online fora. There were few barriers to seeking advice.

**Conclusion:**

Informal, inter-and intra-cadre advice networks existed. Fellow primary healthcare unit staff were preferred, particularly midwives, but networks were not limited to the primary healthcare unit. Additional research is needed to associate network properties with outcomes and pilot network interventions with central actors.

## Background

Social Network Analysis (SNA) is a research approach consisting of a set of theories and methods for mapping communication, information flow and relationships between individuals or groups. SNA has been applied in a wide range of social and physical science fields [1–3]. While some of the seminal SNA articles are health related [4], and there is a growing body of research on health professional advice networks [5–7], little is known about networks among frontline health workers in low and middle-income countries [8–10].

Over the last 10 years, Ethiopia has expanded its health work force considerably, through the introduction of a community-based cadre, health extension workers (HEWs). Yet skilled human resources for health remain a constraint in the equitable availability, accessibility and delivery of healthcare [11]. Ethiopia’s impressive gains in achieving child health targets, with respect to the Millennium Development Goals, have been attributed in part to the Health Extension Programme [12, 13]. Recent data suggest maternal and newborn mortality rates (NMR) are starting to improve after a period of stagnation. Although the 2016 Demographic Health Survey (DHS) indicated progress with a promising drop to 29 from 37 neonatal deaths per 1000 live births, deaths in the first month of life still account for a large proportion of overall child mortality [14]. Similarly, maternal mortality stagnated with no improvement between the 2005 and 2011 DHS reports [15]. The most recent DHS shows a decline to 442 from 676 deaths per 100,000 live births in 2016 [14]. These gains in health outcomes are encouraging and may be attributable to the health systems changes described above. Nevertheless, maternal and neonatal mortality rates are unacceptably high and well away from the Sustainable Development Goals (SDGs) targets for 2030. Ethiopia’s previous maternal and newborn health targets were relative to their levels in 1990; the new SDGs set absolute targets for all countries to meet by 2030 [16, 17]. To achieve these SDGs, Ethiopia must reduce neonatal deaths from 29 to 12 per 1000 live births and maternal deaths from 442 to 70 per 100,000 live births in an environment of increasingly constrained health resources [14, 17, 18].

Communication among healthcare workers could be a critical contextual factor affecting whether or not mothers and newborns receive life-saving interventions. Understanding professional advice networks provides foundational information for designing network-based interventions to improve health outcomes [19]. This study aimed to contribute to the understanding of professional advice networks of frontline health workers in Ethiopia. Specifically, this research explored the properties of professional advice networks; the content of advice exchange, the context in which advice exchange took place; who participated in advice exchange; and the extent to which advice networks met healthcare worker needs. Our study focused on healthcare workers implementing a government program known as Community Based Newborn Care (CBNC), designed to address gaps in maternal and newborn care. This subset of healthcare workers were chosen as they were implementing a new health program, thus creating the possibility of advice exchange related to these services.

### Ethiopian health delivery context

Ethiopia has a decentralised, structured model for the delivery of healthcare with the Federal Ministry of Health as the policy-setting body. The four tiered-system provides services at specialised, zonal and district hospitals and primary healthcare units (PHCUs) [11]. PHCUs serve approximately 25,000 people through a referral health centre and five health posts. In 2003, Ethiopia introduced the Health Extension Program (HEP), an ambitious plan to improve delivery of primary healthcare through the introduction of over 30,000 Health Extension Workers [11]. HEWs are government salaried women, 18 years or older, who completed 10th grade schooling [20]. They work in their kebele, the Ethiopian equivalent of a village, in pairs at a health post after receiving a one-year training to deliver a package of 17 essential preventative and curative health services [11, 21]. HEWs have a formal reporting system whereby within a PHCU all HEWs report to a single designated staff member at the health centre. HEWs are supported by a network of volunteers. Studies of HEWs have found their full potential unrealised, calling for additional training and resources for them to maximise their impact on maternal and newborn health [11, 21–24].

In 2013, the Ethiopian government launched CBNC to build on the HEP platform [25]. CBNC aims to improve newborn health outcomes using a framework of “four Cs” and 9 components, see Additional file 1 [26]. CBNC depends on HEWs working with Woman Health Development Teams to identify and refer sick newborns, and when referral is not possible, treat in the community. Health systems are social systems, in Ethiopia linking the community to health services relies on the professional relationships of HEWs [27].

### Social network analysis

A social network is a set of socially relevant ‘nodes’ representing actors - either individuals or organisations - connected by one or more relations [28]. In SNA, the patterns in the relationships between actors are studied. This is distinct from much quantitative public health research, which looks at the relationship between variables and outcomes of interest [29, 30]. While there is a longstanding history of social network analysis in health, [3] recent reviews suggest that the potential has yet to be realised [10, 31, 32]. Much of the work to date focused on spread of disease, diffusion of ideas, impact of social networks on individual health behaviour and inter-organisational structure of health systems [33]. There was little known about professional communication among health professionals [33]. Research on healthcare worker advice networks has mostly been descriptive and not related to the provision of primary healthcare [7, 34–37].

Applications of SNA methods in the community-based health contexts of low- and middle-income countries (LMIC) are similarly limited [38]. To date there have been few social network analysis studies in Ethiopia [39–43] and none in primary healthcare settings.

Relational data can be collected through questionnaires, interviews, observations and analysis of existing records, diaries or other methods [44]. These data are then populated into matrices, or tables and uploaded into software designed to generate visualisations, knowns as sociograms, and calculate network properties. In SNA, visualising data is both a means of presenting findings as well as a tool for identifying patterns and generating findings. The most basic sociograms depict actors as points with connecting lines representing relationships. It is possible to overlay additional information (“attributes”) to qualify the actors or their relationships. These “attributes” can be displayed by changing the colour, size or shape of the actor. The strength of the relationship can be represented by adjusting the thickness of lines connecting actors [45].

SNA studies are characterised by whether the network is directed or undirected, valued or unvalued, and by how the boundaries are defined [44]. Directed networks capture if the relationship is one-way, where one actor initiates and another receives or two-way where both initiate and receive. In contrast, in undirected networks, the relationship either exists or does not exist. Valued networks quantify the strength of the relationship between actors [46]. Network boundaries can be specified either through what is known as a ‘realist approach’ whereby study participants define their own network boundaries or through a ‘nominalist approach’, which uses formal criteria to determine the network or a hybrid combining the two [47].

The overall objective of this study was to contribute to our understanding of professional advice networks of PHCU staff in rural Ethiopia.

## Methods

### Aims

This research aimed to describe the properties of professional advice networks, the content of advice exchange, the context in which advice exchange took place and the extent to which the existing advice networks met healthcare worker needs.

### Study design

This was an observational, cross-sectional network study. There were two stages of data collection: (i) a structured network survey, followed by (ii) semi-structured qualitative interviews. The structured network survey captured valued, directed networks using a roster of PHCU staff from our selected PHCUs, but allowed for respondents to nominate, or name other “off-roster” healthcare professionals as either having provided or sought advice for each of the advice networks of interest. It was directed and valued, with data such as who provided or sought advice from whom, both within the PHCU and beyond it, and the frequency of their interaction were documented.

#### Sampling

PHCUs in zones implementing CBNC formed the sampling pool. Two PHCUs per agrarian region (Amhara, Southern Nations and National People, Oromia and Tigray) were purposively selected for diversity considering number of health posts, number of healthcare workers and coverage of key maternal and newborn health services.

Semi-structured qualitative interviews were conducted with 20 network study participants to explore and gain an in-depth understanding of patterns emerging from the quantitative analysis. Participants were purposively selected to capture the range of PHCU network properties (densest networks, individuals with highest eigenvector centrality, least off-roster advice exchange, least ties and greatest distance, breadth of cadres and individual network characteristics.

#### Tool design

Preliminary interviews to inform study design took place in 2013, a network tool was pre-tested in April 2015 and again in August 2015, translated and back translated, revised and field tested in November 2015. It captured the frequency of seeking and giving advice among PHCU staff using a roster. The following respondent characteristics, also known as attribute data were also captured: gender, age, cadre (health officer, midwife, health extension worker and all categories of nurses) and total years of experience.

The semi-structured interview guide was initially developed in August 2015, revised following analysis of quantitative data and further refined after the initial qualitative interviews. Themes explored included who is sought for advice and why, reasons for advice exchange, if advice needs are being met, and barriers to advice exchange.

#### Data collection

Data were collected by two research teams after a three-day training. The first PHCU’s data were collected together by the two research teams with the lead researcher overseeing and managing the daily review sessions. Research teams consisted of two interviewers and one supervisor who were native speakers of the languages involved (Amharic, Oromifya or Tigrinya). Data were collected over a period of 3 weeks in November and December 2015.

Semi-structured interviews were conducted in June and August 2016 by two teams of trained qualitative researchers. Teams consisted of an interviewer and an interpreter. Interviews were conducted in private spaces in a mix of English, Amharic, Oromifya or Tigrinia. Interviews were sound recorded and detailed notes were expanded immediately following interviews.

#### Data management

Network survey data were collected using paper forms. All rosters and 160 network questionnaires were double entered into Excel [48]. Discrepancies were reviewed and reconciled. Data were imported into UCINET 6.0 for analysis [49].

Each PHCU had seven network matrices (all advice exchange, all advice giving, all advice seeking, all antenatal care advice exchange, all maternity advice exchange, all postnatal care advice exchange and all newborn care advice exchange) and one table of attribute data.

Qualitative data included 20 sets of expanded field notes written in English. Sound recordings were used to spot check the translation and expanded field notes. These were imported into MAXQDA 10.0 for analysis.

#### Data analysis

For each PHCU, a valued adjacency matrix was prepared in Excel for each of the seven networks. Additional networks were created by collapsing data captured into themes: all ties, all advice seeking and all advice giving. Data were imported into UCINET and dichotomised for the calculation of network and actor-level metrics and then imported into Netdraw for visualisations. See Additional file 2 for definitions of network metrics and their calculations [50]. Final figures were regenerated in InDesign.

Qualitative data were coded based on inductive and deductive approaches, building from grounded theory, but more applied, focusing less on theory development [51, 52]. An initial coding tree, with code definitions based on review of literature, understanding of the subject area and initial readings of the expanded field notes was applied to a subset of interviews, reviewed, revised and then reapplied. Coded text fragments were reviewed by segment and intersections of codes were reviewed. Codes were then grouped by respondent cadre to analyse whether patterns emerged by cadre.

## Results

### Network survey

The eight PHCUs each consisted of one health centre and an average of 4.38 Health posts (SD 1.51) (see Table 1).

**Table 1:** PHCU characteristics

PHCU	Network size	Participant response rate	Number of Health Centres	Number of Health Posts	Total number of Facilities	Network Surveys Administered	Qualitative Interviews Conducted
PHCU A	19	95%	1	5	6	18	5
PHCU B	24	96%	1	3	4	23	0
PHCU C	19	100%	1	4	5	19	0
PHCU D	19	100%	1	4	5	19	5
PHCU E	15	100%	1	2	3	15	5
PHCU F	25	100%	1	5	6	25	0
PHCU G	17	100%	1	5	6	17	5
PHCU H	24	100%	1	7	8	24	0
Mean	20.25	0.99	1.00	4.38	5.38	20.00	2.50
Standard deviation	3.65	0.02	0.00	1.51	1.51	3.59	2.67
**Total**	**162**	**99%**	**8**	**35**	**43**	**160**	**20**

Approximately two thirds of the 160 participants were female, with health officers disproportionately male (88%), and midwives disproportionately female (81%) (Table 2). The average number of years of experience was 3.6, with 2.5 years at their current post. HEWs on average had the longest total experience and most years at their current post. Overall, 46% were trained or orientated in CBNC programme. All HEWs should be trained in CBNC and from this sample of 160, 78% reported being trained.

**Table 2:** Network survey respondent characteristics by cadre

Cadre	Number Male	Number Female	Total Number Respondents	Average Age	Average Years of Experience	Average Years at post
Health Officers	15	2	17	26.0	3.3	1.3
Midwives	4	17	21	23.5	1.9	1.3
Nurses	34	29	63	26.0	3.2	2.0
Health Extension Workers	1	58	59	25.1	4.7	3.8
**Average/total**	**54**	**106**	**160**	**25.5**	**3.6**	**2.5**

### Network metrics

Across all networks and all PHCUs the following average network level metrics were observed: density .26 (SD.11), degree centrality .45 (SD.08), distance 1.94 (SD.26), number of ties 95.63 (SD 35.46), size of network 20.25 (SD 3.65) Table 3 presents the network level statistics by network metric, allowing easy comparison of each network metric across networks and PHCUs. Additional file 3 presents the same data, grouped by PHCU providing an overview of each PHCU by network type. Patterns emerge with typically ANC and maternity advice networks being denser and having more ties than PNC and Newborn care advice networks. Half of the PHCUs had this same pattern across the networks with degree centrality. There were some exceptions: for PHCU E and F, ANC advice exchange was noticeably denser, however maternity, PNC and newborn care advice exchange networks had similar density. The other clear pattern was that certain PHCUs had more advice exchanging than others (PHCUs E and F had many more ties versus PHCUs A and B).

**Table 3:** Network-level SNA Metrics Grouped by Metric

Network-level SNA metrics	All networks (ALL)	All advice seeking networks (AS)	All advice giving networks (AG)	All ANC advice seeking or giving networks (ANC)	All Maternity advice seeking or giving networks (MAT)	All PNC advice seeking or giving networks (PNC)	All newborn care advice seeking or giving networks (Newborn)
**Degree Centrality**							
**PHCU**	**ALL**	**AS**	**AG**	**ANC**	**MAT**	**PNC**	**Newborn**
PHCU A	0.48	0.47	0.58	0.50	0.54	0.43	0.40
PHCU B	0.43	0.40	0.43	0.45	0.34	0.38	0.37
PHCU C	0.40	0.36	0.37	0.37	0.37	0.34	0.46
PHCU D	0.40	0.42	0.36	0.50	0.25	0.27	0.20
PHCU E	0.35	0.44	0.42	0.35	0.46	0.44	0.32
PHCU F	0.49	0.55	0.47	0.50	0.43	0.45	0.51
PHCU G	0.52	0.50	0.47	0.37	0.51	0.20	0.26
PHCU H	0.57	0.38	0.54	0.47	0.45	0.27	0.26
mean	0.45	0.44	0.45	0.44	0.42	0.35	0.35
standard deviation	0.08	0.06	0.08	0.06	0.09	0.09	0.11
**Out Degree Centrality**							
**PHCU**	**ALL**	**AS**	**AG**	**ANC**	**MAT**	**PNC**	**Newborn**
PHCU A	0.31	0.21	0.36	0.22	0.24	0.19	0.21
PHCU B	0.50	0.12	0.50	0.52	0.16	0.41	0.40
PHCU C	0.47	0.30	0.44	0.37	0.31	0.23	0.52
PHCU D	0.35	0.13	0.39	0.24	0.15	0.30	0.17
PHCU E	0.39	0.25	0.64	0.24	0.27	0.53	0.50
PHCU F	0.49	0.20	0.50	0.37	0.28	0.36	0.26
**PHCU**	**ALL**	**AS**	**AG**	**ANC**	**MAT**	**PNC**	**Newborn**
PHCU G	0.43	0.25	0.53	0.34	0.47	0.16	0.23
PHCU H	0.53	0.26	0.60	0.33	0.36	0.26	0.23
mean	0.44	0.22	0.49	0.33	0.28	0.30	0.32
standard deviation	0.08	0.06	0.10	0.10	0.10	0.12	0.14
**In Degree Centrality**							
**PHCU**	**ALL**	**AS**	**AG**	**ANC**	**MAT**	**PNC**	**Newborn**
PHCU A	0.54	0.56	0.18	0.57	0.53	0.43	0.27
PHCU B	0.41	0.48	0.18	0.34	0.39	0.18	0.13
PHCU C	0.35	0.36	0.26	0.31	0.43	0.23	0.23
PHCU D	0.47	0.36	0.45	0.41	0.27	0.18	0.23
PHCU E	0.46	0.63	0.18	0.39	0.49	0.22	0.19
PHCU F	0.58	0.63	0.20	0.45	0.45	0.40	0.43
PHCU G	0.43	0.45	0.13	0.27	0.27	0.09	0.23
PHCU H	0.35	0.45	0.19	0.29	0.36	0.07	0.10
mean	0.45	0.49	0.22	0.38	0.40	0.23	0.23
standard deviation	0.08	0.11	0.10	0.10	0.10	0.13	0.10
**Density**							
**PHCU**	**ALL**	**AS**	**AG**	**ANC**	**MAT**	**PNC**	**Newborn**
PHCU A	0.21	0.13	0.11	0.12	0.11	0.09	0.08
PHCU B	0.17	0.10	0.09	0.11	0.07	0.05	0.05
PHCU C	0.33	0.22	0.20	0.20	0.20	0.12	0.12
PHCU D	0.16	0.10	0.13	0.11	0.08	0.05	0.06
PHCU E	0.50	0.27	0.26	0.28	0.25	0.22	0.25
PHCU F	0.28	0.14	0.19	0.19	0.15	0.15	0.13
PHCU G	0.22	0.14	0.13	0.12	0.12	0.04	0.10
PHCU H	0.18	0.09	0.12	0.11	0.09	0.06	0.04
mean	0.26	0.15	0.15	0.16	0.13	0.10	0.10
standard deviation	0.11	0.06	0.06	0.06	0.06	0.06	0.07
**Number of ties**							
**PHCU**	**ALL**	**AS**	**AG**	**ANC**	**MAT**	**PNC**	**Newborn**
PHCU A	71	45	36	42	37	32	26
PHCU B	94	56	49	61	36	25	28
PHCU C	113	74	68	69	69	41	41
PHCU D	56	35	45	37	26	17	20
PHCU E	104	56	55	58	53	47	52
PHCU F	166	85	111	113	88	92	77
**PHCU**	**ALL**	**AS**	**AG**	**ANC**	**MAT**	**PNC**	**Newborn**
PHCU G	60	39	35	33	33	11	26
PHCU H	101	52	68	63	52	33	21
mean	95.63	55.25	58.38	59.50	49.25	37.25	36.38
standard deviation	35.46	17.02	24.73	25.31	20.85	25.08	19.66
**Distance**							
**PHCU**	**ALL**	**AS**	**AG**	**ANC**	**MAT**	**PNC**	**Newborn**
PHCU A	2.00	2.10	1.50	2.30	2.10	2.10	1.80
PHCU B	2.40	2.30	2.00	2.30	2.00	1.70	1.70
PHCU C	1.80	1.80	2.40	2.30	2.30	1.90	2.00
PHCU D	2.00	2.60	2.60	2.40	2.70	1.40	2.00
PHCU E	1.50	1.60	1.90	2.20	2.30	2.20	2.20
PHCU F	1.80	2.40	2.20	1.90	2.00	2.40	2.40
PHCU G	2.00	2.10	2.20	2.10	2.00	1.40	1.90
PHCU H	2.00	1.70	2.10	2.20	2.10	2.60	2.00
mean	1.94	2.08	2.11	2.21	2.19	1.96	2.00
standard deviation	0.26	0.35	0.33	0.16	0.24	0.44	0.22

#### Actor-level network metrics

In addition to calculating network level properties, actor-level metrics were calculated and the cadre of the actor with the highest value is reported in Additional file 4 for each of the networks. Midwives were far more likely to be the actor with the highest in-degree centrality, meaning the most people within the PHCU came to them for advice. This is highlighted in Additional file 5 and is true for all subject areas, although they were as equally sought as nurses for advice related to providing newborn care.

### Visualisations

**Fig. 1:**
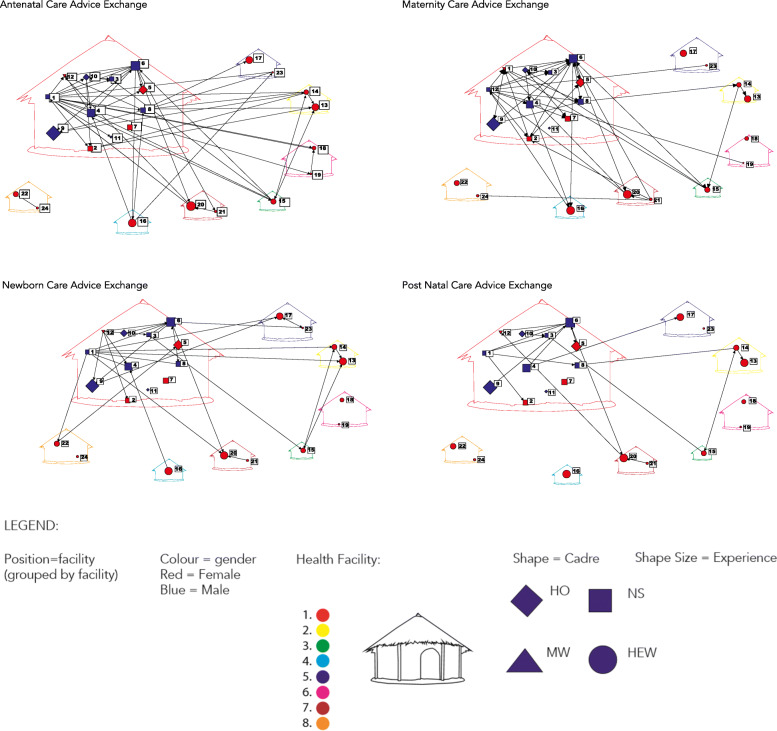
Primary Health Care Unit “H” Dichotomized Sociogram Network

Each PHCU had their networks visualised both with dichotomised data, which facilitated aggregating the ties across networks, and valued data, which added a layer of understanding related to the frequency of interactions. To illustrate the variability within a PHCU across these networks, PHCU H was selected. Figure 1 visualises four dichotomised networks with advice seeking and giving for each care area aggregated into one. These sociograms show more ties and fewer isolates for ANC and maternity advice networks relative to PNC and newborn care advice networks.

**Fig. 2:**
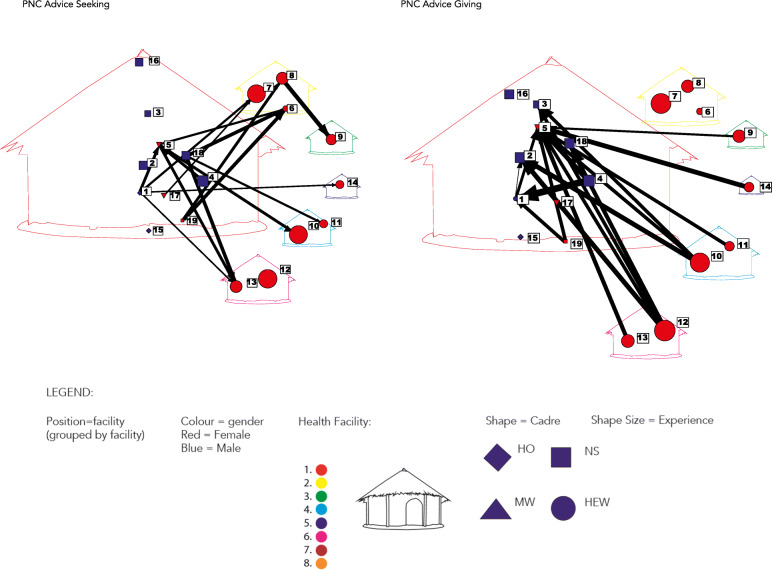
Primary Health Care Unit “A” Valued Postnatal Care Advice Seeking and Giving

Valued data were visualised in the same way (with respect to the node attribute data) as for the other sociograms with the exception that the line widths reflect the frequency of interaction (thicker lines reflecting greater frequency ranging from daily to yearly). PHCU A’s PNC advice seeking and advice giving sociograms were selected to illustrate in Fig. 2 how there seems to be more individuals seeking advice than giving advice.

**Fig. 3:**
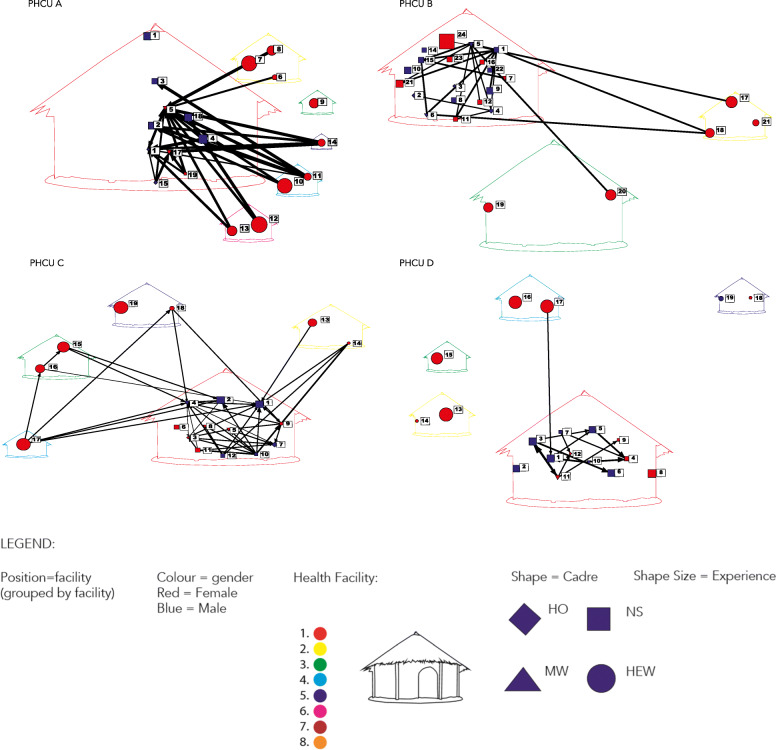
Valued Maternity Advice Seeking Across Primary Health Care Unit A, B, C and D

**Fig. 4:**
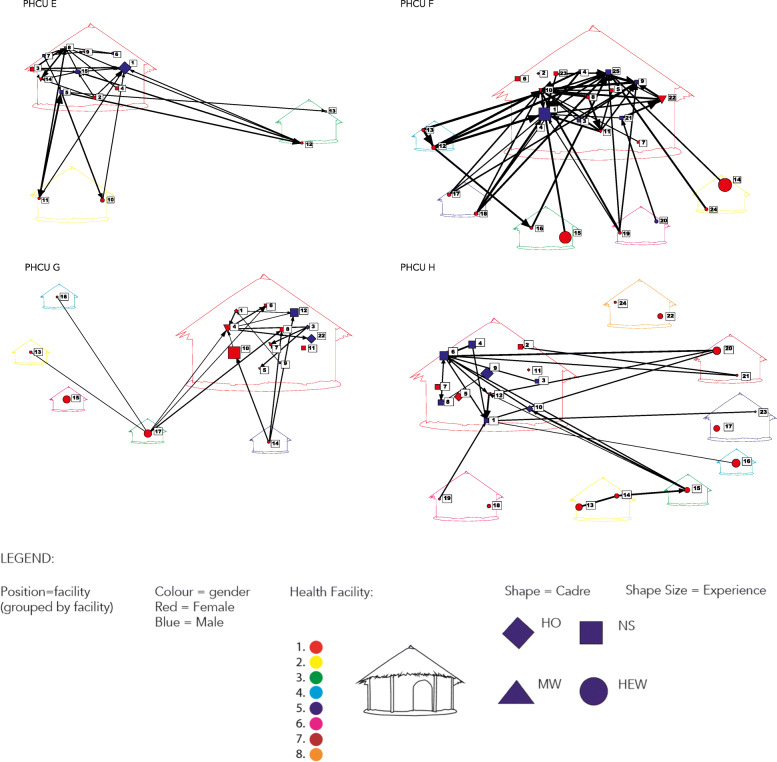
Valued Maternity Advice Seeking for Primary Health Care Unit E, F, G and H

To show the variability across the PHCUs for a given type of network, maternity advice seeking was selected to show across all 8 PHCUs in Figs. 3 and 4. For all of these graphs midwives play a central role, as expected, despite some variability. In Fig. 3, PHCU D has only advice exchange happening at the HC with the exception of one HEW engaged, whereas in Fig. 4, PHCU F has many HEWs engaged frequently with HC staff and even some HEWs seeking advice from each other. The intra and intercadre advice exchange depicted by PHCU F was more typical of the findings across PHCUs and topics for advice exchange. The data on formal supervisory structures were available only from a subset of those PHCUs that were selected for the qualitative inquiry. Due to staff turnover, only one could be analysed along with the quantitative network data. This example confirmed what was observed across other PHCUs, a willingness to engage in informal advice exchange outside of formal supervisory structures.

#### Off roster advice seeking

Of the four cadres of healthcare workers, health officers reported the fewest number of individuals they either sought or gave advice to who were not working within their PHCU. However, after adjusting for the different number of HCWs per cadre those distinctions largely disappear. For these HCWs more advice is sought off roster than they are giving to those outside of the PHCU. This is particularly the case for Health officers and HEWs. By far the most off roster advice exchange occurred for nurses and HEWs seeking advice regarding providing ANC.

### Qualitative findings

#### Who is sought for advice and why?

Reasons for going to a specific person for advice were typically because of that specific person’s training and knowledge, less so because of their years of experience. One healthcare worker noted that because of their training in integrated community case management, PHCU colleagues seek their guidance. Some respondents said that they relied on formal supervisory structures, however they appeared relatively infrequent with most people describing qualities of the individuals’ knowledge and skills dictating their advice seeking behaviour rather than just formal structures. The examples of consulting supervisors related to situations where they “had some fear or discomfort with the situation and didn’t want to take accountability for something going wrong.” [Health officer, Tigray] Personality or level of comfort with the person was mentioned as a secondary factor that contributed to who was approached for advice.

In general, current PHCU colleagues were sought for advice, however if they were not available, former classmates and colleagues were the most common individuals sought for advice. For example, one respondent said he’d first go to experienced people in his PHCU, but if they are unable to give advice he would:

“call some peers, people who [I] went to school with and grew up with, working in other HCs or hospitals who have several years of experience, or even professors to ask for advice in complicated cases.” [Health Officer, Amhara]

This was consistent across cadres: health officers, nurses and HEWs for both routine and urgent questions. A nurse in Tigray described a case of postpartum haemorrhage when his supervisor was away at a training, so he had called a midwife he had previously worked with who was now at a different health centre. Several people within this PHCU mentioned seeking advice from this same midwife who had been transferred.

The furthest afield anyone mentioned seeking advice from was from a friend in Addis Ababa because colleagues within the PHCU did not know how to handle the situation. Only one person mentioned seeking advice from someone outside of the PHCU because of not being comfortable asking for a colleague’s advice. This does not appear to be a widespread concern for most healthcare workers.

#### Reasons for advice exchange

According to our respondents, the range of advice given on providing ANC care included many topics already covered in their training. One nurse explained that they need repetition because learning the content theoretically is so different from doing it practically. This was also the case for advice exchange around other service delivery areas.

“While the integrated community case management manual is very clear it seems that [the HEW I was advising] lacked confidence and contacted me at the health centre for reassurance.” [Nurse, Tigray]

While there are examples of advice being sought for providing antenatal care, it is noteworthy that many respondents said they felt comfortable providing ANC and believed they did not need advice. Several healthcare workers mentioned fearing deliveries and that those with less experience sought those with more for reassurance and guidance. A HEW described seeking advice from another HEW because she had referred more women to the health centre for delivery and she wondered what methods she was using that might be helpful in her own work.

#### Are advice needs being met?

All interviewees indicated that they had always been able to have their specific questions answered when seeking advice. Interviewers probed further, asking if there were ever situations in which they were unable to have their questions answered, and heard adamant statements from several respondents: “how would I treat them if I [still] needed advice?” [HEW, Amhara] and “[I] will not let uncertainties rest until [I] get [my] questions answered.” [HEW, Tigray].

No interviewees said they were unable to get the advice they needed, although some described asking more than one person or consulting other resources. This could reflect a response bias, an unwillingness to admit to providing care while having questions about providing that care. Or it could be that when they had a clear question they could generally find an answer. However, they only asked when they were aware that they did not know something. While their specific questions were addressed, many described a desire for additional training, because as one respondent said, “I will benefit from additional information I am not aware of” [Midwife, Oromia]

#### Barriers to advice exchange

Many of the HEWs said that they do not deliver babies, although as the closest HCWs to the community they are often involved in referral to the health centre. Some HEWs said they are not involved in postnatal care, although others said they are involved just for identifying dangers signs and referral.^1^ When asked why people do not seek advice from her, a HEW said “the HC staff don’t ask because they are at a higher level of knowledge, education and training than me and that they would ask each other. They wouldn’t think to ask a HEW.” [HEW, Amhara].

Respondents commented on the logistical constraints in seeking advice. The poor mobile phone network in some rural areas was mentioned as a barrier, particularly in one PHCU’s catchment area, which had only gained network access within the last 8 months. “it [the mobile network] had affected it [advice seeking] before and I remedied this by handling the case to the best of [my] knowledge and asking later to clarify what [I] had done.”[Health Officer, Amhara].

Respondents commented that workload sometimes interfered with seeking advice for non-urgent cases. Advice exchange for these non-urgent situations typically happened in person in either at ad-hoc or routine meetings. If urgent, [from respondent’s perspective] mobile phones were used, particularly for HCWs seeking advice from more skilled providers at the health centre or woreda.

“[I] usually ask for advice by phone, especially in the case of emergencies and this advice seeking comes whenever a difficult case arises, once or twice a month. There is nothing stopping [me] from asking for advice as long as the phone networks are working. The network rarely fails around the HC so this is not a big hurdle “[Health Officer, Tigray].

One HEW described her fellow HEW as being “intimidated easily to ask questions, so [I] served as a conduit.” [HEW, Amhara] Another said that “If I do not know the answer, I would call someone who is not here, does not work in this place.” [Midwife, Oromia] These were the only examples given even with probing with hypothetical reasons for why someone might not feel comfortable seeking advice from their colleagues within the PHCU. Another mentioned language as a possible barrier and that they “could access advice more easily” if rather than speaking in Amharic, they spoke in a local language as “this would avoid missing out on any information.”

## Discussion

Professional advice networks present an opportunity to more effectively change health provider practice than mere training as healthcare workers are more likely to be convinced by trusted colleagues. This is particularly relevant in resource constrained settings where funding for and feasibility of training everyone does not exist. This study explores existing advice networks in the context of a new program introduction to learn about these networks and see if there may be potential for harnessing them for future program needs.

There are no standards for appropriate frequency of advice exchange among healthcare workers in Ethiopia or elsewhere. Presumably most healthcare workers should have some advice needs or if not, should be the source of advice for their colleagues, particularly in the context of a new programme being introduced for which not all have been trained. Further complicating interpreting these data is that network scientists debate what network properties constitute a “healthy network” [5].

There were no previous studies of PHCU healthcare worker professional advice networks documenting network properties. Neither were there previous studies of the same healthcare professionals comparing their professional advice exchange networks for antenatal, maternity, postnatal or newborn care provision. Therefore, this study contributes a foundation which future studies can use to compare their findings.

The advice networks observed had few isolates with limited distance between actors. Five PHCUs had low density and high centrality. Taken together these network properties can be interpreted that most providers are participating in advice exchange and that typically there are actors that serve as hubs. There is diversity across the PHCUs in terms of network properties, with variability across all metrics. While some PHCUs fit a pattern whereby there are more ties for antenatal and maternity advice networks, other network properties and other PHCUs were more nuanced.

Of note is the willingness of healthcare workers to seek advice and the lack of rigid adherence to the supervisory structure. Given the Ethiopian healthcare delivery context is very hierarchical the existence of informal advice networks is a noteworthy finding. Similarly, a study of Dutch nurses in a long-term care facility found advice networks to be non-hierarchical, although they only looked within one cadre [53]. In studies of inter-professional communication hierarchy is common [7, 35, 54–57]. That said, the reliance on informal advice networks may be a product of infrequent supportive supervision visits and review meetings.

Advice exchange varied by cadre, but universally there was more advice exchanging between cadres than within cadres. This runs counter to what has been found in some western contexts, where professional homophily runs high and advice is primarily sought within a cadre [7, 35, 58]. The direction was typically from the cadre with less training to that with more, with midwives being more engaged for maternity care advice and health officers more for newborn care advice needs. It is important to note that there is specialisation and division of labour within the health centre and between different cadres which could explain some of the patterns observed in the advice seeking. While HEWs say they are not sought by other cadres for advice because of knowledge differentials, the subtext could very well be that a HEW’s advice is not sought by other cadres because of power and hierarchy. Advice exchanged between cadres often took place over mobile phones if it was urgent. If it was not urgent, they would wait for supervisory visits or routine meetings. This preference for informal, in person communication is consistent with findings among other, albeit western healthcare contexts [58, 59].

It is a significant finding that the advice networks are largely meeting the advice needs of healthcare workers. Barriers to advice exchange included poor phone networks and knowing when to ask for advice. Advice needs are being met only in those situations where healthcare workers know what they do not know and seek advice. While a preference was expressed for in person communication, evaluations of the HEP note that routine PHCU meetings and supportive supervision fall short of standards [24]. The lack of regular supervision may also account for why these supervisors were not featured more prominently in these advice networks.

Professional advice networks of PHCU staff prioritise proximity, but are not restricted to it. This is relevant both within the PHCU and to those working outside the PHCU. Another study in Italy found geographic distance a factor in physician advice networks [60]. The individuals who were not PHCU staff sought for advice ranged from peers from training programs, former colleagues who have since been transferred to those working in nearby PHCUs or hospitals. Typically, they were engaged only if colleagues within the PHCU were unavailable or in one case could not answer the question. This suggests that the personal connection may matter less as the established relationships are not prioritized as a first stop for advice.

This study shows the value of combining quantitative network methods with qualitative inquiry. This is an approach that more network studies may consider applying should their research objectives include understanding the context surrounding network ties. Such complementary methods could strengthen proposed role of SNA in program implementation [5].

There is a need for further research to understand why there is more advice exchanging around providing antenatal and childbirth care than providing postnatal and newborn care. The aim is to be able to answer the following questions: is this pattern a reflection of true information needs, a product of more patients engaging with the health system for those services, or a reflection of knowledge gaps in providing postnatal and newborn care sufficient enough for providers not to be able to identify their knowledge limitations. Answering these questions will help identify the appropriate policy response. This could be additional pre-service training on postnatal and newborn care such that once in the field healthcare workers are primed to “know what they don’t know.”

This study’s findings, while foundational, could have relevant policy implications for the Ethiopian Federal Ministry of Health and other LMIC contexts. Language was mentioned as a barrier in some advice exchange and some providers described relying on guidelines and reference materials, if these materials could be available in local languages this would be an easy-to-implement “quick win”. These findings suggest that healthcare workers value training and knowledge over years of experience when seeking advice. Therefore it may be possible to achieve better outcomes through focusing training on specific individuals within a PHCU whose primary role could be sharing knowledge. Additional research would be needed to test such a concept and see if this targeted training approach yields at least equivalent learning across PHCU staff and patient outcomes.

Additionally, these findings point towards the potential of cadre-based targeted in-service trainings with more central figures within informal advice networks. Further studies, building on what has been done elsewhere exploring the value of network-based training models [61] would be needed to pilot both the feasibility and measure the impact of such an approach to ensure the intervention is scalable and that equivalent outcomes are achieved.

Strengthening supervisory structures may enable them to be more commonly used fora for advice exchange. Health centre staff were noticeably transient relative to HEWs, this may affect advice networks. Further research is needed to understand the directionality of this affect and the policy implications.

### Limitations

Approximately 8 months passed between the collection of the quantitative data and the collection of the qualitative data. Recall bias may have compromised data quality, but this should be minimal given the study asked about hypothetical situations and the most recent example of advice exchange. The qualitative data are not explicitly linked to the quantitative network data as the time periods of reflection are inherently different and staff changes were noted. While there were anticipated advantages to analysing the quantitative network data and using that data to select participants for the qualitative study, future mixed methods network studies may consider conducting the qualitative interviews in parallel with the network surveys.

While effort was made to limit misunderstandings around the type of communication of interest through use of a scripted description to clarify and standardise meaning of “advice seeking and advice giving”, it remains possible that study respondents did not have a common understanding.

## Conclusions

This exploratory study provides foundational information regarding professional advice networks of PHCU healthcare workers in Ethiopia. This study establishes that PHCU staff involved in delivery of maternal and newborn health services have informal advice networks outside of supervisory structures. Advice exchanges occurred between cadres and used both face to face meetings and mobile phones to exchange advice. More research is needed to understand if the patterns in advice exchange across antenatal, maternity, postnatal and newborn care accurately reflected advice needs or if they reflected a bias towards antenatal and maternity care knowledge and thus individuals being better placed to self-identify knowledge gaps in those areas. Fellow PHCU staff were prioritised but networks were not limited to those within their geographic area. Policy implications include focusing future training on cadres more central in advice networks, such as midwives for antenatal, maternity and postnatal care and nurses or health officers for newborn care. One possibility could be training an individual or two per PHCU to be the “knowledge sharing focal persons”, who attend trainings and are responsible for sharing learnings. Another could be cadre-based in service trainings with the same mandate for sharing learnings. Further studies would be needed to pilot such approaches to ensure achievement of equivalent learning and patient outcomes. A simple policy implication of this work could be providing guidelines and reference material in local languages. Additional research is needed to more accurately measure performance to link network properties to patient outcomes as well as investigate the impact of turnover and absence on advice networks, ideally through a longitudinal network study. This study demonstrates the feasibility of using social network analysis methods in rural Ethiopia, which has implications for other African and low or middle-income countries. This study also shows the value of combining quantitative network methods with qualitative research to lend a greater understanding of network properties. Mixed SNA method studies should be used more widely in these contexts as they provide a different lens and understanding of professional advice networks in settings where resources for health are increasingly constrained and as such networks may be an efficient and effective way to change practice.

## Supplementary information

**Additional file 1.** Community based Newborn Care Framework. Description of data: graphic detailing the “4 C”‘s and the “9 Components” of Community based Newborn Care

**Additional file 2.** Social Network Analysis Network Metric Definitions and Formulas. Description of data: Table with text describing the actor and network level SNA metrics’ definitions, calculations and pathway to their output in UCINET

**Additional file 3.** Network level metrics grouped by PHCU. Description of data: Table with numeric data (whole numbers and decimals) reflecting values of the following network level metrics for each PHCU: degree centrality, out degree, in degree, density and number of ties. Theses metrics are presented for each of the following networks: all, all advice seeking, all advice giving, all antenatal care, all maternity care, all postnatal care and all newborn care

**Additional file 4.** Actor-level metrics within each PHCU and network type: Cadre of actor with highest value. Description of data: Table with numeric data (whole numbers) of actor-level network metrics reflecting cadre of actor with highest value within that PHCU.

**Additional file 5.** Cadre with the highest in degree centrality value by network. Description of data: Table with numeric data (whole numbers) reflecting number of networks where each cadre has the highest in degree centrality.

## Data Availability

The datasets generated and/or analysed during the current study are not publicly available due to the possibility of identifying individuals who participated but are available from the corresponding author on reasonable request.

## Abbreviations

AG: Advice Giving
ANC: Antenatal Care
AS: Advice Seeking
CBNC: Community Based Newborn Care
HC: Health Centre
HEP: Health Extension Programme
HEW: Health Extension Worker
HO: Health officer
HP: Health Post
iCCM: integrated Care Case Management
LMIC: Low and middle income country
MAT: Maternity care
MW: Midwife
NEW: Newborn care
NS: Nurse
PHCU: Primary healthcare unit
PNC: Postnatal care
SNA: Social Network Analysis
SNNP: Southern National Nationalities Peoples (Region)
WHDT: Women’s Health Development Team

## Footnotes

1 This is unusual. Postnatal care is explicitly part of the HEW package of services. However, they may have interpreted “care” to mean providing clinical care, in which case they refer women to health centres.
