# Supplementary material for: Professional advice for primary healthcare workers in Ethiopia: a social network analysis
Source: BMC Health Serv Res. 2020 Jun 17;20:551. doi: 10.1186/s12913-020-05367-3 (PMC7302001; doi:10.1186/s12913-020-05367-3)
Supplement: Supplementary file 1 — Additional file 1. Community based Newborn Care Framework. Description of data: graphic detailing the “4 C”‘s and the “9 Components” of Community based Newborn Care [file 12913_2020_5367_MOESM1_ESM.pdf]

Addition File 1: Community Based Newborn Care Framework

4 "C"s

1. early prenatal and postnatal **Contact** with the mother and newborn;
2. **Case-identification** of newborns with signs of possible severe bacterial infection;
3. **Care,** or treatment that is appropriate and initiated as early as possible; and
4. **Completion** of a full seven-day course of appropriate antibiotics.

9 components

1. Early identification of pregnancy
2. Provision of focused antenatal care (FANC)
3. Promotion of institutional delivery
4. Safe and clean delivery
5. Provision of immediate newborn care, including chlorhexidine cord care
6. Recognition of asphyxia, initial stimulation and resuscitation of the newborn baby
7. Prevention and management of hypothermia
8. Management of pre-term and low birth weight neonates and
9. Management of neonatal sepsis and very severe disease (VSD) at community level
