# Supplementary material for: Professional advice for primary healthcare workers in Ethiopia: a social network analysis
Source: BMC Health Serv Res. 2020 Jun 17;20:551. doi: 10.1186/s12913-020-05367-3 (PMC7302001; doi:10.1186/s12913-020-05367-3)
Supplement: Supplementary file 2 — Additional file 2. Social Network Analysis Network Metric Definitions and Formulas. Description of data: Table with text describing the actor and network level SNA metrics’ definitions, calculations and pathway to their output in UCINET [file 12913_2020_5367_MOESM2_ESM.docx]

| Additional File 2: Social Network Analysis Network Metric Definitions and Formulas | | | |
| --- | --- | --- | --- |
| **Network Metric** | **Definition** | **Calculation/Formula** | **UCINET** |
| **Actor- level** | | | |
| Betweeness Centrality | The number of geodesic paths (the shortest path between two actors) that pass through a given actor. | There are different ways of calculating betweeness centrality, for our purposes we used Freeman's Betweeness Centrality. As described by Analytic technologies: "More precisely, if gij is the number of geodesic paths from i to j and gikj is the number of paths from i to j that pass through k, then gikj/gij is the proportion of geodesic paths from i to j that pass through k. The sum ck = gikj/gij for all i,j pairs is betweenness centrality. Normalized betweenness divides simple betweenness by its maximum value." | Network>Centrality and Power>Freemans Betweeness> Node Betweeness Output report look at actor with the highest value, report nBetweeness (normalized). |
| Out Degree | In a directed network, out degree centrality is the number or ratio of ties a given actor nominates, or initiates a direct relation towards another actor. | It can be expressed either as a whole number or a ratio. As a whole number it is the sum of outward directed ties to other actors. As a ratio, this sum is then divided by the total number of possible ties (n-1), the number of actors within the network, minus 1 (as the actor in question cannot have a tie with itself) | Network>Centrality>Degree Output report provides two tables. The first table provides actor level Out Degree centrality in first column, OutDegree as a whole number, and as a normalized percentage in the third column under NrmOutDegree |
| **Network Metric** | **Definition** | **Calculation/Formula** | **UCINET** |
| In Degree | In a directed network, in degree centrality is the number or ration of ties a given actor receives from other actors within the network | It can be expressed either as a whole number or a ratio. As a whole number it is the sum of all ties initiated from other actors. As a ratio, this sum is then divided by the total number of possible ties (n-1) | Network>Centrality>Degree Output report provides two tables. The first table provides actor level In Degree centrality in the second column, InDegree as a whole number, and as a normalized percentage in the fourth column under NrmInDegree |
| Eigenvector centrality | This is a weighted centrality metric. As with most centrality metrics it reflects the number of ties between an actor and the other actors within the network. | With Eigenvector centrality, the centrality of actors directly connected to the actor is taken into account. Those connected to more central actors have higher eigenvector centrality metrics. According to Analytic Technologies: Eigenvector centrality "is like recursive version of degree centrality: Start by assigning centrality score of 1 to all nodes (vi = 1 for all i), Recompute scores of each node as weighted sum of centralities of all nodes in a node's neighborhood: vi = xijvj, Normalize v by dividing each value by the largest value, Repeat steps ii and iii until values of v stop changing." | Network>centrality>eigenvector Output report node(s) with the highest values, as these are the nodes who along with their alters’ ties are the most connected. |
| **Network Metric** | **Definition** | **Calculation/Formula** | **UCINET** |
| **Network-level** | | | |
| Centralization | Degree Centralization captures direct, or incident ties to other actors at the network level | It is expressed as a ratio or a percentage. To find network degree centralization, you must find the most central actor (C*). Then calculate the variation in degree centrality to the other network actors (Ci) *and sum the differences: Σ(C*-Ci), then divide this by the largest possible degree centralization, Max Σ(C*-Ci). Formula: Σ(C*-Ci) / Max Σ(C*-Ci) From UCSD ppt "network measures web" | Network>Centrality>Degree UCINET Report graph centralization scores at end of output |
| Out Degree Centralization | In a directed network, the ratio of ties actors’ nominate, or initiate towards other actors out of those possible | It is expressed as a ratio or percentage. All outward directed ties are summed, then divided by the total number of possible ties (the number of actors within the network) | Network>Centrality>Degree (old) UCINET Report Network centralization (outdegree) percentage for each matrix following the actor level table and the descriptive statistics table |
| In Degree Centralization | In a directed network, the ratio of ties actors receive from other actors out of those possible | It is expressed as a ratio or percentage. All inward directed ties are summed, then divided by the total number of possible ties (the number of actors within the network) | Network>Centrality>Degree (old)UCINET Report Network centralization (indegree) percentage for each matrix following the actor level table and the descriptive statistics table |
| **Network Metric** | **Definition** | **Calculation/Formula** | **UCINET** |
| **Network-level** | | | |
| Density | Density of a network is the total number of relational ties divided by the total possible number of relational ties. | total number of relational ties divided by the total possible number of relational ties d=L/ [n(n-1)/2], when L equals the actual number of ties (Prell p.167) | Network>Cohesion>Density (overall) Output report first column (density) |
| Number of Ties | Total number of ties between all actors in a network. | Sum of all ties between all actors (dichotomized)within a network | Network>Cohesion>Density (overall) Output report second column (No. of ties) |
| Distance | Length of optimal path between actors in a network | Average length of optimal path between actors in a network | Network>Cohesion>Geodesic Distances  Output report average value |
| Isolates | An actor with no ties to other actors within the network | The number of actors without any ties for a given network are totalled | N/A |
