# Supplementary material for: Professional advice for primary healthcare workers in Ethiopia: a social network analysis
Source: BMC Health Serv Res. 2020 Jun 17;20:551. doi: 10.1186/s12913-020-05367-3 (PMC7302001; doi:10.1186/s12913-020-05367-3)
Supplement: Supplementary file 3 — Additional file 3. Network level metrics grouped by PHCU. Description of data: Table with numeric data (whole numbers and decimals) reflecting values of the following network level metrics for each PHCU: degree centrality, out degree, in degree, density and number of ties. Theses metrics are presented for each of the following networks: all, all advice seeking, all advice giving, all antenatal care, all maternity care, all postnatal care and all newborn care [file 12913_2020_5367_MOESM3_ESM.docx]

| **Additional File 3: Network level metrics grouped by PHCU** | | | | | | | | | |
| --- | --- | --- | --- | --- | --- | --- | --- | --- | --- |
| **PHCU** | **All networks (ALL)** | **All advice seeking networks (AS)** | **All advice giving networks (AG)** | **All ANC advice seeking or giving networks (ANC)** | **All Maternity advice seeking or giving networks (MAT)** | **All PNC advice seeking or giving networks (PNC)** | **All newborn care advice seeking or giving networks (Newborn)** | **descriptive statistics** | |
| **PHCU A** | **ALL** | **AS** | **AG** | **ANC** | **Maternity** | **PNC** | **Newborn** | **mean** | **standard deviation** |
| Degree Centrality | 0.48 | 0.47 | 0.58 | 0.50 | 0.54 | 0.43 | 0.40 | 0.49 | 0.06 |
| Out Degree | 0.31 | 0.21 | 0.36 | 0.22 | 0.24 | 0.19 | 0.21 | 0.25 | 0.06 |
| In Degree | 0.54 | 0.56 | 0.18 | 0.57 | 0.53 | 0.43 | 0.27 | 0.44 | 0.157 |
| Density | 0.21 | 0.13 | 0.11 | 0.12 | 0.11 | 0.09 | 0.08 | 0.12 | 0.043 |
| No. ties | 71 | 45 | 36 | 42 | 37 | 32 | 26 | 41.29 | 14.51 |
| Distance | 2 | 2.1 | 1.5 | 2.3 | 2.1 | 2.1 | 1.8 | 1.99 | 0.261 |
| **PHCU B** | **ALL** | **AS** | **AG** | **ANC** | **Maternity** | **PNC** | **Newborn** | **mean** | **standard deviation** |
| Degree Centrality | 0.43 | 0.40 | 0.43 | 0.45 | 0.34 | 0.38 | 0.37 | 0.40 | 0.04 |
| Out Degree | 0.50 | 0.12 | 0.50 | 0.52 | 0.16 | 0.41 | 0.40 | 0.37 | 0.166 |
| In Degree | 0.41 | 0.48 | 0.18 | 0.34 | 0.39 | 0.18 | 0.13 | 0.30 | 0.138 |
| Density | 0.17 | 0.10 | 0.09 | 0.11 | 0.07 | 0.05 | 0.05 | 0.09 | 0.043 |
| No. ties | 94 | 56 | 49 | 61 | 36 | 25 | 28 | 49.86 | 23.8 |
| Distance | 2.4 | 2.3 | 2 | 2.3 | 2 | 1.7 | 1.7 | 2.06 | 0.288 |
| **PHCU** | **All networks (ALL)** | **All advice seeking networks (AS)** | **All advice giving networks (AG)** | **All ANC advice seeking or giving networks (ANC)** | **All Maternity advice seeking or giving networks (MAT)** | **All PNC advice seeking or giving networks (PNC)** | **All newborn care advice seeking or giving networks (Newborn)** | **descriptive statistics** | |
| **PHCU C** | **ALL** | **AS** | **AG** | **ANC** | **Maternity** | **PNC** | **Newborn** | **mean** | **standard deviation** |
| Degree Centrality | 0.40 | 0.36 | 0.37 | 0.37 | 0.37 | 0.34 | 0.46 | 0.38 | 0.04 |
| Out Degree | 0.47 | 0.30 | 0.44 | 0.37 | 0.31 | 0.23 | 0.52 | 0.38 | 0.104 |
| In Degree | 0.35 | 0.36 | 0.26 | 0.31 | 0.43 | 0.23 | 0.23 | 0.31 | 0.078 |
| Density | 0.33 | 0.22 | 0.20 | 0.20 | 0.20 | 0.12 | 0.12 | 0.20 | 0.071 |
| No. ties | 113 | 74 | 68 | 69 | 69 | 41 | 41 | 67.86 | 24.22 |
| Distance | 1.8 | 1.8 | 2.4 | 2.3 | 2.3 | 1.9 | 2 | 2.07 | 0.256 |
| **PHCU D** | **ALL** | **AS** | **AG** | **ANC** | **Maternity** | **PNC** | **Newborn** | **mean** | **standard deviation** |
| Degree Centrality | 0.40 | 0.42 | 0.36 | 0.50 | 0.25 | 0.27 | 0.20 | 0.34 | 0.107 |
| Out Degree | 0.35 | 0.13 | 0.39 | 0.24 | 0.15 | 0.30 | 0.17 | 0.25 | 0.103 |
| In Degree | 0.47 | 0.36 | 0.45 | 0.41 | 0.27 | 0.18 | 0.23 | 0.34 | 0.113 |
| Density | 0.16 | 0.10 | 0.13 | 0.11 | 0.08 | 0.05 | 0.06 | 0.10 | 0.041 |
| No. ties | 56 | 35 | 45 | 37 | 26 | 17 | 20 | 33.71 | 13.92 |
| Distance | 2 | 2.6 | 2.6 | 2.4 | 2.7 | 1.4 | 2 | 2.24 | 0.469 |
| **PHCU E** | **ALL** | **AS** | **AG** | **ANC** | **Maternity** | **PNC** | **Newborn** | **mean** | **standard deviation** |
| Degree Centrality | 0.35 | 0.44 | 0.42 | 0.35 | 0.46 | 0.44 | 0.32 | 0.40 | 0.055 |
| **PHCU** | **All networks (ALL)** | **All advice seeking networks (AS)** | **All advice giving networks (AG)** | **All ANC advice seeking or giving networks (ANC)** | **All Maternity advice seeking or giving networks (MAT)** | **All PNC advice seeking or giving networks (PNC)** | **All newborn care advice seeking or giving networks (Newborn)** | **descriptive statistics** | |
| **PHCU E** | **ALL** | **AS** | **AG** | **ANC** | **Maternity** | **PNC** | **Newborn** | **mean** | **standard deviation** |
| Out Degree | 0.39 | 0.25 | 0.64 | 0.24 | 0.27 | 0.53 | 0.50 | 0.40 | 0.157 |
| In Degree | 0.46 | 0.63 | 0.18 | 0.39 | 0.49 | 0.22 | 0.19 | 0.37 | 0.175 |
| Density | 0.50 | 0.27 | 0.26 | 0.28 | 0.25 | 0.22 | 0.25 | 0.29 | 0.092 |
| No. ties | 104 | 56 | 55 | 58 | 53 | 47 | 52 | 60.71 | 19.41 |
| Distance | 1.5 | 1.6 | 1.9 | 2.2 | 2.3 | 2.2 | 2.2 | 1.99 | 0.324 |
| **PHCU F** | **ALL** | **AS** | **AG** | **ANC** | **Maternity** | **PNC** | **Newborn** | **mean** | **standard deviation** |
| Degree Centrality | 0.49 | 0.55 | 0.47 | 0.50 | 0.43 | 0.45 | 0.51 | 0.49 | 0.038 |
| Out Degree | 0.49 | 0.20 | 0.50 | 0.37 | 0.28 | 0.36 | 0.26 | 0.35 | 0.115 |
| In Degree | 0.58 | 0.63 | 0.20 | 0.45 | 0.45 | 0.40 | 0.43 | 0.45 | 0.139 |
| Density | 0.28 | 0.14 | 0.19 | 0.19 | 0.15 | 0.15 | 0.13 | 0.17 | 0.05 |
| No. ties | 166 | 85 | 111 | 113 | 88 | 92 | 77 | 104.57 | 30.17 |
| Distance | 1.8 | 2.4 | 2.2 | 1.9 | 2 | 2.4 | 2.4 | 2.16 | 0.257 |
| **PHCU G** | **ALL** | **AS** | **AG** | **ANC** | **Maternity** | **PNC** | **Newborn** | **mean** | **standard deviation** |
| Degree Centrality | 0.52 | 0.50 | 0.47 | 0.37 | 0.51 | 0.20 | 0.26 | 0.40 | 0.13 |
| Out Degree | 0.43 | 0.25 | 0.53 | 0.34 | 0.47 | 0.16 | 0.23 | 0.34 | 0.138 |
| **PHCU** | **All networks (ALL)** | **All advice seeking networks (AS)** | **All advice giving networks (AG)** | **All ANC advice seeking or giving networks (ANC)** | **All Maternity advice seeking or giving networks (MAT)** | **All PNC advice seeking or giving networks (PNC)** | **All newborn care advice seeking or giving networks (Newborn)** | **descriptive statistics** | |
| **PHCU G** | **ALL** | **AS** | **AG** | **ANC** | **Maternity** | **PNC** | **Newborn** | **mean** | **standard deviation** |
| In Degree | 0.43 | 0.45 | 0.13 | 0.27 | 0.27 | 0.09 | 0.23 | 0.27 | 0.135 |
| Density | 0.22 | 0.14 | 0.13 | 0.12 | 0.12 | 0.04 | 0.10 | 0.12 | 0.054 |
| No. ties | 60 | 39 | 35 | 33 | 33 | 11 | 26 | 33.86 | 14.7 |
| Distance | 2 | 2.1 | 2.2 | 2.1 | 2 | 1.4 | 1.9 | 1.96 | 0.264 |
| **PHCU H** | **ALL** | **AS** | **AG** | **ANC** | **Maternity** | **PNC** | **Newborn** | **mean** | **standard deviation** |
| Degree Centrality | 0.57 | 0.38 | 0.54 | 0.47 | 0.45 | 0.27 | 0.26 | 0.42 | 0.123 |
| Out Degree | 0.53 | 0.26 | 0.60 | 0.33 | 0.36 | 0.26 | 0.23 | 0.37 | 0.143 |
| In Degree | 0.35 | 0.45 | 0.19 | 0.29 | 0.36 | 0.07 | 0.10 | 0.26 | 0.141 |
| Density | 0.18 | 0.09 | 0.12 | 0.11 | 0.09 | 0.06 | 0.04 | 0.10 | 0.047 |
| No. ties | 101 | 52 | 68 | 63 | 52 | 33 | 21 | 55.71 | 25.83 |
| Distance | 2 | 1.7 | 2.1 | 2.2 | 2.1 | 2.6 | 2 | 2.10 | 0.271 |
