# Supplementary material for: Professional advice for primary healthcare workers in Ethiopia: a social network analysis
Source: BMC Health Serv Res. 2020 Jun 17;20:551. doi: 10.1186/s12913-020-05367-3 (PMC7302001; doi:10.1186/s12913-020-05367-3)
Supplement: Supplementary file 4 — Additional file 4. Actor-level metrics within each PHCU and network type: Cadre of actor with highest value. Description of data: Table with numeric data (whole numbers) of actor-level network metrics reflecting cadre of actor with highest value within that PHCU. [file 12913_2020_5367_MOESM4_ESM.docx]

**Additional File 4: Actor-level metrics within each PHCU and network type: Cadre of actor with highest value**

| **PHCU** | **Cadre and value of actor with the highest value SNA metric for each PHCU and network captured  (Cadre abbreviations NS=nurse, MW=midwife, HEW=health extension worker, HO=health officer)** | | | | | | |
| --- | --- | --- | --- | --- | --- | --- | --- |
|  | **All networks (ALL)** | **All advice seeking networks (AS)** | **All advice-giving networks (AG)** | **All ANC advice seeking or giving networks (ANC)** | **All Maternity advice seeking or giving networks (MAT)** | **All PNC advice seeking or giving networks (PNC)** | **All newborn care advice seeking or giving networks (New-born)** |
| **Betweeness** | | | | | | | |
| **PHCU** | **ALL** | **AS** | **AG** | **ANC** | **MAT** | **PNC** | **Newborn** |
| PHCU A | MW (32.616) | HO (12.963) | MW (8.932) | MW (27.451) | MW (30.065) | MW (20.261) | HO (12.418) |
| PHCU B | MW (34.314) | MW (25.395) | MW (6.456) | NS (24.776) | MW (8.992) | MW (3.162) | MW (2.964) |
| PHCU C | NS (16.256) | NS (13.533) | NS (16.966) | MW (26.747) | MW (24.599) | NS (11.492) | NS (11.928) |
| PHCU D | NS (20.937) | MW (13.426) | NS (36.029) | MW (24.946) | MW (16.176) | NS(1.961) | NS (3.595) |
| PHCU E | HO (18.656) | NS (17.582) | NS (14.698) | NS (21.361) | NS (39.011) | MW (26.377) | NS (36.364) |
| PHCU F | NS (19.201) | MW (16.757) | MW (12.530) | NS (18.916) | NS (15.854) | NS (27.926) | NS (31.908) |
| PHCU G | MW (39.701) | HO (14.958) | MW (29.722) | MW (22.361) | MW (30.625) | HEW (2.5) | MW (13.125) |
| PHCU H | NS (27.803) | NS (8.696) | NS (16.614) | NS (23.142) | NS (20.487) | NS (11.133) | NS (6.719) |
| **Out Degree Centrality** | | | | | | | |
| **PHCU** | **ALL** | **AS** | **AG** | **ANC** | **MAT** | **PNC** | **Newborn** |
| PHCU A | MW (9) | HEW (6) | MW (8) | HO (6) | NS (6) | HO (5) | HO/HEW(5) |
| **PHCU** | **Cadre and value of actor with the highest value SNA metric for each PHCU and network captured  (Cadre abbreviations NS=nurse, MW=midwife, HEW=health extension worker, HO=health officer)** | | | | | | |
|  | **All networks (ALL)** | **All advice seeking networks (AS)** | **All advice-giving networks (AG)** | **All ANC advice seeking or giving networks (ANC)** | **All Maternity advice seeking or giving networks (MAT)** | **All PNC advice seeking or giving networks (PNC)** | **All new-born care advice seeking or giving networks (New-born)** |
| **Out Degree Centrality (contd.)** | | | | | | | |
| **PHCU** | **ALL** | **AS** | **AG** | **ANC** | **MAT** | **PNC** | **New-born** |
| PHCU B | HO (13) | HO (13) | NS (6) | MW (10) | MW/NS (5) | MW (5) | NS (4) |
| PHCU C | NS (14) | NS (9) | NS (11) | NS (10) | NS (9) | NS (6) | NS (11) |
| PHCU D | NS (9) | NS/MW/MW (4) | NS (9) | MW (6) | NS (4) | NS (6) | NS (4) |
| PHCU E | HO (12) | NS (7) | HO (12) | NS (7) | HO (7) | NS (10) | NS (10) |
| PHCU F | NS (18) | MW/HEW (8) | NS/MW (16) | NS (13) | MW (10) | MW (12) | NS (9) |
| PHCU G | MW (10) | HO/HEW (6) | MW (10) | HEW (7) | MW (9) | HO/HEW (3) | HEW (5) |
| PHCU H | NS (16) | HEW (8) | NS (16) | NS (10) | NS (10) | NS (7) | NS (6) |
| **In Degree Centrality** | | | | | | | |
| **PHCU** | **ALL** | **AS** | **AG** | **ANC** | **MAT** | **PNC** | **Newborn** |
| PHCU A | MW (13) | MW (12) | MW (5) | MW (12) | MW (11) | MW (9) | HO/MW (6) |
| PHCU B | NS (15) | MW/NS (5) | NS (13) | NS (14) | HO (10) | NS (10) | NS (10) |
| PHCU C | MW (12) | MW (10) | MW (8) | MW (9) | MW (11) | MW (6) | MW (6) |
| **PHCU** | **Cadre and value of actor with the highest value SNA metric for each PHCU and network captured  (Cadre abbreviations NS=nurse, MW=midwife, HEW=health extension worker, HO=health officer)** | | | | | | |
|  | **All networks (ALL)** | **All advice seeking networks (AS)** | **All advice-giving networks (AG)** | **All ANC advice seeking or giving networks (ANC)** | **All Maternity advice seeking or giving networks (MAT)** | **All PNC advice seeking or giving networks (PNC)** | **All new-born care advice seeking or giving networks (Newborn)** |
| **In Degree Centrality (contd.)** | | | | | | | |
| **PHCU** | **ALL** | **AS** | **AG** | **ANC** | **MAT** | **PNC** | **Newborn** |
| PHCU D | MW (11) | MW (8) | MW (10) | MW (9) | MW(6) | MW (4) | MW (5) |
| PHCU E | HO (13) | HO (12) | HEW (6) | HO (9) | MW (10) | HO (6) | NS/HEW (6) |
| PHCU F | MW (20) | MW (18) | NS/MW (9) | MW (15) | MW (14) | MW (13) | NS (13) |
| PHCU G | MW (10) | MW (9) | HO/MW (4) | MW (6) | MW (6) | MW/HEW (2) | MW (5) |
| PHCU H | NS (12) | NS (12) | MW (7) | NS (9) | NS (10) | NS/HO/HEW (3) | NS (3) |
| **Eigenvector Centrality** | | | | | | | |
| **PHCU** | **ALL** | **AS** | **AG** | **ANC** | **MAT** | **PNC** | **Newborn** |
| PHCU A | MW (40.4%) | HO (43.9%) | MW (54.3%) | MW (47.6%) | MW (50.6%) | MW (51.5%) | HO (53.2%) |
| PHCU B | MW (37.3%) | MW (40.9%) | NS (43.3%) | MW (40.4%) | HO (44.6%) | NS (55.6%) | NS (55.4%) |
| PHCU C | NS (33.9%) | NS (34.2%) | NS (36.9%) | NS (40.4%) | NS (35.1%) | NS (44.7%) | NS (46.8%) |
| PHCU D | MW (42.3%) | MW (48.7%) | MW (42.1%) | NS (38.8%) | NS (46.2%) | NS (54.8%) | MW (51.2%) |
| PHCU E | HO (34.8%) | HO (39%) | HO (39.1%) | HO (39.4%) | HO (41.4%) | NS (42.8%) | NS (38.8%) |
| **PHCU** | **Cadre and value of actor with the highest value SNA metric for each PHCU and network captured  (Cadre abbreviations NS=nurse, MW=midwife, HEW=health extension worker, HO=health officer)** | | | | | | |
|  | **All networks (ALL)** | **All advice seeking networks (AS)** | **All advice-giving networks (AG)** | **All ANC advice seeking or giving networks (ANC)** | **All Maternity advice seeking or giving networks (MAT)** | **All PNC advice seeking or giving networks (PNC)** | **All new-born care advice seeking or giving networks (Newborn)** |
| **Eigenvector Centrality (contd.)** | | | | | | | |
| **PHCU** | **ALL** | **AS** | **AG** | **ANC** | **MAT** | **PNC** | **Newborn** |
| PHCU F | NS (32.7%) | NS (38.7%) | MW (34.8%) | NS (37.2%) | NS (39.8%) | NS (36.6%) | NS (41.8%) |
| PHCU G | MW (46.8%) | MW (50%) | MW (49.8%) | MW/HEW (45.4%) | MW (58.8%) | HEW (56.9%) | MW (51.1%) |
| PHCU H | NS (42.0%) | NS (43.7%) | NS (44.7%) | NS (45.8%) | NS (49.8%) | NS (48.3%) | NS (59.9%) |
