# Supplementary material for: Professional advice for primary healthcare workers in Ethiopia: a social network analysis
Source: BMC Health Serv Res. 2020 Jun 17;20:551. doi: 10.1186/s12913-020-05367-3 (PMC7302001; doi:10.1186/s12913-020-05367-3)
Supplement: Supplementary file 5 — Additional file 5. Cadre with the highest in degree centrality value by network. Description of data: Table with numeric data (whole numbers) reflecting number of networks where each cadre has the highest in degree centrality. [file 12913_2020_5367_MOESM5_ESM.docx]

| **Additional File 5 Cadre with the highest in degree centrality value by network** | | | | | |
| --- | --- | --- | --- | --- | --- |
| **Cadre** | **ANC** | **Maternity** | **PNC** | **Newborn** | **Total** |
| Health Officer | 1 | 1 | 2 | 1 | 5 |
| Midwife | 5 | 6 | 5 | 4 | 20 |
| Nurse | 2 | 1 | 2 | 4 | 9 |
| Health Extension Worker | 0 | 0 | 2 | 1 | 3 |
